# *Lacticaseibacillus casei* CNCM I-5663 supplementation maintained muscle mass in a model of frail rodents

**DOI:** 10.3389/fnut.2022.928798

**Published:** 2022-08-10

**Authors:** Muriel Giron, Muriel Thomas, Marianne Jarzaguet, Camille Mayeur, Gladys Ferrere, Marie-Louise Noordine, Stéphanie Bornes, Dominique Dardevet, Christophe Chassard, Isabelle Savary-Auzeloux

**Affiliations:** ^1^INRAE, UMR 1019, Unité de Nutrition Humaine, Université Clermont Auvergne, Clermont-Ferrand, France; ^2^Université Paris-Saclay, INRAE UMR 1319, AgroParisTech, Micalis Institute, Jouy-en-Josas, France; ^3^INRAE UMR 0545, Unité Mixte de Recherche sur le Fromage, Université Clermont Auvergne, VetAgro Sup, Aurillac, France

**Keywords:** muscle, sarcopenia, probiotic, lactic acid bacteria, insulin sensitivity, protein synthesis

## Abstract

The aim of this study was to identify a probiotic-based strategy for maintaining muscle anabolism in the elderly. In previous research, we found that individuals experiencing short bowel syndrome (SBS) after an intestinal resection displayed beneficial metabolic adjustments that were mediated by their gut microbes. Thus, these bacteria could potentially be used to elicit similar positive effects in elderly people, who often have low food intake and thus develop sarcopenia. Gut bacterial strains from an SBS patient were evaluated for their ability to (1) maintain *Caenorhabditis elegans* survival and muscle structure and (2) promote protein anabolism in a model of frail rodents (18-month-old rats on a food-restricted diet: 75% of *ad libitum* consumption). We screened a first set of bacteria in *C. elegans* and selected two *Lacticaseibacillus casei* strains (62 and 63) for further testing in the rat model. We had four experimental groups: control rats on an *ad libitum* diet (AL); non-supplemented rats on the food-restricted diet (R); and two sets of food-restricted rats that received a daily supplement of one of the strains (∼10^9^ CFU; R+62 and R+63). We measured lean mass, protein metabolism, insulin resistance, cecal short-chain fatty acids (SCFAs), and SCFA receptor expression in the gut. Food restriction led to decreased muscle mass [−10% vs. AL (*p* < 0.05)]. Supplementation with strain 63 tempered this effect [−2% vs. AL (*p* > 0.1)]. The mechanism appeared to be the stimulation of the insulin-sensitive p-S6/S6 and p-eIF2α/eIF2α ratios, which were similar in the R+63 and AL groups (*p* > 0.1) but lower in the R group (*p* < 0.05). We hypothesize that greater SCFA receptor sensitivity in the R+63 group promoted gut-muscle cross talk [GPR41: +40% and GPR43: +47% vs. R (*p* < 0.05)]. Hence, strain 63 could be used in association with other nutritional strategies and exercise regimes to limit sarcopenia in frail elderly people.

## Introduction

Sarcopenia is an age-related decline in muscle mass and strength. It is namely characterized by a loss of leg mass (1–2% per year) and strength (1.5–5% per year) in people older than 50 years (1). The result is an increased risk of falls, hospitalization, and a state of frailty/dependence, making sarcopenia a major public health issue (2, 3). This condition partly develops because, over time, skeletal muscle has a less-effective anabolic response to food ingestion [i.e., resistance to insulin and other anabolic signals, such as leucine (4)] and experiences chronic low-grade inflammation (“inflammageing”) (5). Strategies based on protein/amino acid supplementation, sometimes paired with physical exercise, have been found to help counter the effects of sarcopenia (6, 7). However, these approaches are not always effective and could be more difficult to promulgate in older adults experiencing a loss of appetite or difficulties in exercising (2). It is therefore essential to develop innovative techniques that are better suited to the needs of frail elderly people and that could be combined with other strategies to boost their effectiveness.

Over the past 30 years, research on gut microbiota has greatly expanded, with many studies focusing on how gut microbes and host metabolism interact (8–10). It has been known for years that altered gut microbiota composition and functioning occur in tandem with various metabolic diseases and forms of insulin resistance (11, 12). Furthermore, gut microbiota composition is often imbalanced (i.e., dysbiosis) in older adults, especially in those who are frail and/or who have been institutionalized (13, 14). Compared to healthier individuals, these populations frequently have lower microbial diversity, larger numbers of opportunistic pathogens, and lower levels of species that produce anti-inflammatory and insulin-sensitizing metabolites (e.g., *Faecalibacterium prausnitzii*) (14, 15). Recent studies have found that the gut microbiota and skeletal muscle engage in cross-talk (5, 16–20), which leads us to hypothesize that strategies targeting gut community composition and function could help frail elderly individuals improve their skeletal muscle health. Indeed, some probiotics have been demonstrated to modulate inflammation, metabolic issues (e.g., age-related declines in insulin sensitivity), and muscle function in juvenile rodents (21–24), adult rodents (25), and humans of various ages (26–28) [for a review, see Giron et al. (29)]. The benefits of probiotics have also been investigated using rodent models of aging [SAMP-8 (30–32) and D-galactose (33)] and animal models of cachexia (34). However, researchers have rarely used healthy older rodents (35) and have never used “frail” rodents, such as older, food-restricted rats.

The elderly often struggle to achieve healthy levels of food intake, which suggests that complementary treatment strategies should focus on boosting the absorption of any nutrients consumed. Interestingly, research has shown that individuals who developed short bowel syndrome (SBS) following intestinal resection displayed metabolic adjustments several months later: better nutrient absorption in the intestine and the effective metabolic use of nutrients (36–38). Mechanistically, these changes could be partially related to the microbiota. Indeed, people with SBS experience drastic changes in their gut communities, where the microbiota is 90% lactobacilli (36). Moreover, when the microbiota of a human with SBS was transferred to axenic rats, the latter showed increased levels of GLP-1 and ghrelin in their plasma, peptides known to improve insulin sensitivity and appetite regulation (37, 39). These findings, therefore, suggest that strategies involving probiotic supplementation could promote insulin sensitivity, metabolic efficiency, and, possibly, muscle protein anabolism in frail elderly people.

## Materials and methods

### Isolation and identification of lactic acid bacteria strains

#### Strain isolation

We collected feces from a germ-free rat inoculated with fecal microbiota from an SBS patient 30 days after the inoculation had taken place (37). The fecal sample displayed a stable microbial composition similar to that of the inoculum. In an anaerobic chamber, 250 mg of the fecal sample was diluted with a rich, non-selective BHI-S medium. The mixture was spread on Petri dishes and incubated at 37°C for 48 h under anaerobic conditions (GENBox Jar^®^ and GENBag anaer^®^; Biomerieux, France). After the incubation period, at least three different colony types were observed. Sixty-one colonies were used to inoculate De Man, Rogosa, and Sharpe (MRS) broth and were then incubated at 37°C for 24 h under anaerobic conditions. Gram staining and microscopic observations showed that the bacterial cultures were pure, bacilliform, and Gram-positive. These cultures were placed in glycerol (final concentration: 16%) and frozen at −80°C. Aliquots of these cultures were centrifuged (10 min at 4,000 *g*), and the resulting pellets were stored at −20°C until molecular identification could occur. In tandem, the strains were visualized using scanning electron microscopy (MIMA2, INRAE, Jouy-en-Josas, France). To this end, culture samples were centrifuged (10 min at 4,000 *g*). The resulting pellets were resuspended in a fixative agent (glutaraldehyde sodium cacodylate buffer) and left at room temperature overnight before the observations.

#### Strain identification

The DNA in the pellets was extracted using Godon’s method (40). It was first screened using semi-quantitative real-time PCR and the specific primers for genus *Lactobacillus* described by Mayeur et al. (41). Then, we sequenced the isolates identified as lactic acid bacteria (LAB)/*Lactobacillus*: we amplified 16S rDNA *via* PCR with the universal primers E8-F and U1492-R and the intermediate primers U519-F, U926-R, and U1053-F (Table 1).

**TABLE 1:** Universal and intermediate primers used in 16S rDNA amplification.

Primers	Sequence (5′-3′)
*E8-F*	AGAGTTTGATCCTGG CTCAG
*U1492-R*	ACGGTTACCTTGTTACGACTT
*U519-F*	CAGCMGCCGCGGTAATWC
*U926-R*	CCGTCAATTCCTTTRAGTTT
*U1053-F*	GCATGGCYGYCGTCAG

The PCR mix was composed of 10.4 μl of H_2_O, 4 μl of 5 × Phusion HF Buffer, 0.4 μl of dNTP (10 mM), 1 μl of each primer (10 μM), and 0.2 μl of Phusion High Fidelity Taq Polymerase (2 U/μl) (Thermo Fischer Scientific). Next, 20 μl of the mix and 1 μl of pure bacterial DNA were deposited in a 96-well PCR plate. The amplification cycle consisted of an initial 1-min denaturation step at 98 C, which was followed by 35 cycles of 10 s at 98°C, 30 s at 55°C, and 30 s at 72°C; there was a final 1-min extension step at 72°C (T100™ Thermal Cycler, Bio-Rad). The amplification results were verified *via* electrophoresis (1% agarose gel + 1X GelRed^®^). We used SmartLadder Sample^®^ MW-1700-SA (Eurogentec, France) to confirm molecular weight. The PCR products were sequenced by the Eurofins Genomics platform (Île-de-France, France), and the nucleotide sequences were analyzed using NCBI BLAST^®^ software and GenBank, resulting in the identification of known sequences (>98%).

### Strain growth conditions

All the LAB strains were stored at −80°C in their culture media with 16% glycerol (final concentration). They were then grown overnight at 37°C in MRS broth (Biokar Diagnostics). To monitor their growth dynamics, cultures of each strain were normalized to reach an optical density (OD) of 0.1, and 200-μl samples were placed in a 96-well plate. The experiments were performed at 37°C under aerobic conditions, which were mimicked by sealing the wells with paraffin oil. Samples were analyzed using a Spectra FLUOR Plus Microplate Fluorescence Reader (Tecan Life Sciences, Switzerland), which monitors microbial growth by measuring culture turbidity (i.e., OD at 600 nm) in the liquid growth medium. The experiments were run for 24 h, and turbidity was measured every 30 min. The two strains used in this work, *Lacticaseibacillus casei* 62 and 63, have been deposited in the Institut Pasteur’s collection of microorganisms and cell cultures (reference numbers CNCM-I5662 and CNCM-I5663, respectively; patent application number FR2106084).

### *Caenorhabditis elegans* rearing and synchronization

For our *Caenorhabditis elegans* strains, we used the CGC:N2 wild-type Bristol isolate and PJ1277 [ccIs4251 (Pmyo-3:Ngfp-lacZ; Pmyo-3:Mtgfp) I; him-8 (e1489) IV] (42). The strains were reared at 20°C on a nematode growth medium [plates of agar containing 4 g/L of yeast extract (NGMY)] that was seeded with *Escherichia coli* OP50 as described elsewhere (43). Our *E*. *coli* OP50 strain was obtained from the Caenorhabditis Genetics Center (Minneapolis, MN, United States) and grown overnight at 37°C in Luria broth (Fischer Scientific) the day before the experiment was conducted. During synchronization, eggs and gravid adults were washed with M9 buffer and centrifuged at 1,500 rpm for 2 min (Rotofix 32a, Hettich). The pellet was resuspended in 5 ml of worm bleach (2.5 mL of M9 buffer, 1.5 ml of pure sodium hypochlorite, and 1 ml of 5M sodium hydroxide) and mixed vigorously for 6 min until the bodies of the adult nematodes were disrupted. The action of the sodium hypochlorite was stopped using 20 ml of M9 buffer, and the mixture was centrifuged at 1,500 rpm for 2 min. The pellet containing the eggs was washed twice with M9 buffer and then resuspended in 20 ml of M9 buffer. Finally, the eggs were incubated at room temperature for 24 h using a tube rotator (10 rpm; Boekel Scientific). The resulting L1 larvae were transferred to an NGMY medium seeded with *E. coli* OP50 and then incubated for about 48 h (i.e., until the nematodes reached the L4/adult stage).

#### *Caenorhabditis elegans* survival

The synchronized L4 worms were transferred to an NGMY medium containing 0.12 mM 5-fluorodeoxyuridine FUdR (Sigma, Saint-Louis, United States) in 6-well plates; there were about 20 worms per well. The plates had been seeded 24 h earlier with bacteria strains (∼10^9^ CFU per well): the control group was given *E. coli* OP50, one experimental group was given *L. casei* 62, and another experimental group was given *L. casei* 63. These groups are hereafter referred to as OP50, LAB62, and LAB63. There were three wells for each group, and the plates were incubated at 20°C. The number of living nematodes was counted daily until mortality reached 100%. A nematode was considered to be dead if it failed to respond to gentle movement. This assay was performed three separate times.

#### *Caenorhabditis elegans* muscle structure

Thanks to green fluorescent protein (GFP) labeling, we were able to visualize mitochondria in the nematodes’ body wall muscle *in vivo*. In this way, we could qualitatively assess the structure of the mitochondrial network using a method described elsewhere (44–46). All the images were captured using a fluorescence microscope (100× magnification; Evos^®^ FL, Invitrogen). About 20 worms were visualized at a time. Mitochondrial network structure was categorized as follows: (1) “tubular”: networks were mostly long and interconnected; (2) “intermediate”: interconnected networks occurred in tandem with some smaller fragmented mitochondria; and (3) “fragmented”: the presence of sparse, small, and round mitochondria (44). Four different researchers carried out blind data analysis. They determined the percentages of tubular, intermediate, and fragmented networks over time (adult worms after 1, 4, 7, 11, and 13 days) and across groups (OP50, LAB63, or LAB62).

#### Lactic acid bacteria ingestion by *Caenorhabditis elegans*

Lactic acid bacteria strains that had been cultured overnight were centrifuged (4,000 rpm for 10 min), and the resulting pellet was rinsed three times with M9 buffer. Then, the pellet was placed in 5 ml of M9 buffer + 10% BODIPY staining solution (50 μg/mL of BODIPY™ in DMSO) and allowed to sit for 15 min. The resulting stained bacteria were rinsed twice, added to 5 ml of M9 buffer, and spread on a plate containing NGMY medium. A few nematodes were then placed on the plate. After 24 h, the nematodes’ intestines were examined using a fluorescence microscope (Evos^®^ FL, Invitrogen) to determine if the bacteria had been ingested.

### The model of frailty in rodent

This work was approved by the Animal Care and Use Committee of Auvergne (CEMEA Auvergne; Permit Number: C2EA-02) and the French Ministry of Higher Education, Research, and Innovation (# 20374-2019060714134169V3).

#### Effects of probiotic supplementation on rat insulin resistance and body composition

**FIGURE 1:**
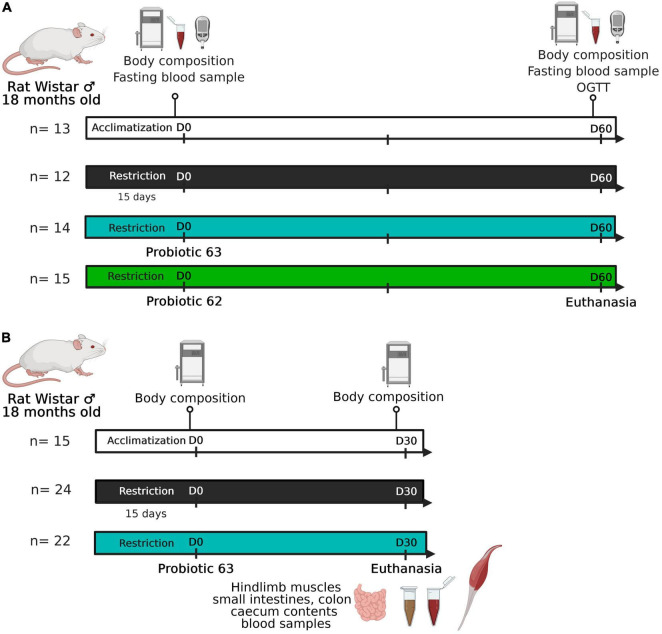
Experimental design. **(A)** Experiment #1: 18-month-old Wistar rats (*n* = 41) experienced a food-restricted diet (75% of *ad libitum* consumption). Starting on day 0, two groups of rats (*n* = 29) began receiving a probiotic supplement (strain 62 or 63; 10^9^ CFU/day). Rat body composition and glucose tolerance were characterized at the beginning (day 0) and end (day 60) of the experiment. **(B)** Experiment #2: 18-month-old Wistar rats (*n* = 46) experienced a food-restricted diet (75% of *ad libitum* consumption). Starting on day 0, half of the food-restricted animals received strain 63 as a probiotic supplement (10^9^ CFU/day). Rat body composition was characterized at the beginning (day 0) and end (day 30) of the experiment. The rodents were then euthanized after one last meal, and tissue samples were taken (i.e., hindlimb muscles, ileum, colon, cecal contents, and blood).

A 2-month experiment was carried out using 18-month-old male Wistar rats (Janvier Labs, France). The rats were housed individually, kept under controlled conditions (22°C, 12:12 light-dark cycle), and fed pellets *ad libitum* (D05, SAFE, Augy, France). During the experiment, all the rats had *ad libitum* access to water. After an initial 2-week acclimation period, the rats were randomly assigned to one of the two groups: a control group, which had *ad libitum* access to food (AL, *n* = 13), and a food-restricted group (R, *n* = 41), which was given 75% of food levels consumed under AL conditions. This first experimental phase lasted 15 days. Then, rats in the R group were randomly assigned to one of the three subgroups: a control food-restricted group (R, *n* = 12), a food-restricted group that was given *L. casei* 62 as a supplement (R+62, *n* = 15), and a food-restricted group that was given *L. casei* 63 as a supplement (R+63, *n* = 14) (Figure 1A). The treatment supplements (10^9^ CFU) and the control supplement (phosphate-buffered saline) were freshly prepared each day and added to 1 g of feed pellets, which were completely consumed by the rats within 15 min. The rats then received their daily allotment of food. This second experimental phase lasted 60 days. We measured food consumption daily and body mass three times a week.

Rat body composition was assessed using magnetic resonance imaging (EchoMRI 4-in-1 system, EchoMRI, Houston, TX, United States) at the beginning (day 0) and the end (day 60) of the second experimental phase. An oral glucose tolerance test (OGTT) was also carried out at the end of the second experimental phase (day 60): rats were fasted for 16 h and then force-fed a glucose solution (dose: 1 g/kg BM) (Figure 1A). We took blood samples from the tail vein (anticoagulant: 200 mM EDTA) when rats were in the fasting state and then 15, 30, 60, 90, and 120 min after the administration of glucose. The plasma samples were frozen in liquid nitrogen and stored at −80°C until the glucose and insulin analyses could be performed. Glucose levels were measured using GOD-PAP diagnostic reagent (BIOLABO SAS, France), and insulin levels were measured using a commercial ELISA kit in accordance with the manufacturer’s instructions (Mercodia, Sweden). Each sample was analyzed in duplicate. The values of the insulin resistance index (HOMA-IR) were calculated as follows: fasting blood insulin (mU/L) × fasting blood glucose (mM)/22.5 as determined from the tail blood samples on days 0 and 60.

#### Effects of probiotic supplementation on rat intestinal physiology and muscle

A second experiment was performed that again used 18-month-old male Wistar rats (Janvier Labs, France) (Figure 1B). The design was similar to that of the first experiment, except that only the LAB strain showing the most promising results was tested (AL, *n* = 15; R, *n* = 24; R+63, *n* = 22). As there was less measurement-related variability in the second experiment, it lasted 1 month instead of 2 months. By shortening the treatment duration, we limited animal mortality and loss of muscle mass. Food consumption was measured daily, and body mass was measured three times a week. Body composition was assessed *via* MRI before the beginning of the study (day −3) and on day 25.

At the end of the second experiment, the animals were euthanized using isoflurane (Figure 1B). An abdominal incision was made, and blood samples were taken from the hepatic vein, portal vein, and abdominal aorta (anticoagulants: EDTA or heparin). The blood was used in biochemical assays (Supplementary Figure 1) that enzymatically measured glucose, lactate, urea, HDL cholesterol, LDL cholesterol, and total cholesterol concentrations using commercial kits and an automated clinical chemistry analyzer (ABX Pentra 400; Horiba Medical, France). We rapidly removed the hindlimb muscles (gastrocnemius, soleus, tibialis anterior, and extensor digitorum longus). They were weighed, freeze-clamped in liquid nitrogen, and then stored at −80°C. Different parts of the intestines (i.e., the ileum and proximal colon) were cleaned, weighed, freeze-clamped in liquid nitrogen, and then stored at −80°C. The contents of the cecum were removed and stored at −80°C for later analysis.

### Tissue analysis

#### Gene expression

##### RNA extraction and reverse transcription

We isolated total RNA from 50 mg of ileum/colon tissue and 100 mg of gastrocnemius tissue using TRIzol Reagent (Invitrogen) and an RNeasy^®^ Mini Kit (Qiagen) in accordance with the manufacturer’s instructions. RNA concentrations and purity were verified using a NanoDrop spectrophotometer (ND-1000, NanoDrop Technologies Inc., United States); RNA quality (1 μg) was verified using electrophoresis (1% agarose gel and ethidium bromide). cDNA was synthesized from 2 μg of DNase-treated RNA (ezDNase, Invitrogen, Thermo Fischer Scientific) using a High-Capacity cDNA Reverse Transcription Kit (Applied Biosystems, Thermo Fischer Scientific) in accordance with the manufacturer’s instructions.

##### Semiquantitative real-time PCR

The cDNA was diluted at 1:50 in RNAse-free water. Real-time quantitative PCR (RT-qPCR) was performed using a Bio-Rad CFX-96 detection system and quantitative qPCR SYBR Green reagents. A total of 10 μl of 2X Power SYBR^®^ Green Master Mix (Applied Biosystems, Thermo Fisher Scientific) was mixed with 1 μl of both forward and reverse primers (10 μM) and 5 μl of diluted cDNA (final volume: 20 μL). The result was incubated at 95°C for 10 min to activate the polymerase. The primer sequences are provided in Table 2. All the PCRs were performed in duplicate under the following conditions: 40 cycles of 95°C for 15 s and 60°C for 45 s. The relative levels of the target genes were normalized using the mean expression of 36B4, Hprt1, and Ywhaz as a reference. Relative gene expression was quantified using the 2^–ΔΔCT^ method.

**TABLE 2:** Real-time quantitative PCR primers used in analyses of rat muscle, ileum, and colon tissue.

Gene	Forward sequence (5′-3′)	Reverse sequence (5′-3′)
**Reference genes**		
36B4	TTCCTAGAGGGTGTCCGCAAT	GCAACAGTCGGGTAGCCAAT
Hprt1	GCAGACTTTGCTTTCCTTGG	TCCACTTTCGCTGATGACAC
Ywhaz	TTGAGCAGAAGACGGAAGGT	CCTCAGCCAAGTAGCGGTAG
**Gastrocnemius muscle**		
Murf-1	AATGCTCCAGTCGGCCCCTG	AGCCCCGAACACCTTGCACA
Atrogin-1	ATCCAGATCAGCAGGCCGGC	CACATGCAGGTCTGGGGCTG
ATG16L	AGGAAGAGGCACGCGACTTG	GCCCTCTCTCTACGCTCGTT
LC3B	CCGGAGCTTCGAACAAAGAG	CAGCTGCTTCTCACCCTTGT
Cathepsin L	GGTGGGGCCTATTTCTGTTG	TCGAGGTCCTTGCTGCTACA
**Ileum and colon**		
GPR43	CTGCTGCCCTTCCGGATCGTG	CAGGCCACCAGAGCAGCGAT
GPR41	GCTCTCCAACACTCTGCATCTGT	GCGACGCAGCTTGTTCACGA

36B4, acidic ribosomal phosphoprotein P0; Hprt1, hypoxanthine-guanine phosphoribosyl transferase 1; Ywhaz, tyrosine 3-monooxygenase/tryptophan 5-monooxygenase activation protein zeta; Murf-1, muscle RING-finger protein-1; LC3B, microtubule-associated protein 1 light chain 3 beta; Atg 16L, autophagy related 16-like 1; GPR43, G-protein coupled receptor 43; GPR41, G-protein coupled receptor 41.

#### Western blot

The state of the proteins S6 and eIF2α (i.e., phosphorylated/dephosphorylated) was assessed to estimate the activation level of the mTOR signaling pathway, which is involved in protein synthesis. To homogenize the fibrous muscle of the gastrocnemius, the tissues were sprinkled with liquid nitrogen in a ball mill (Dangoumeau, Prolabo, Paris, France) as described elsewhere (47). Briefly, a 50-mg aliquot of frozen gastrocnemius powder was homogenized in 10 volumes of lysis buffer. The homogenate was centrifuged at 10,000 *g* for 10 min at 4°C. The protein concentrations were determined using the BCA method (Pierce™ BCA Protein Assay Kit). Protein samples (16 μg) were diluted in sample buffer; separated using precast SDS-PAGE (Mini-PROTEAN TGX Precast Gels, BIO-RAD, France); and transferred to a PVDF membrane (Trans-Blot^®^ Turbo™ Midi-size PVDF, BIO-RAD, France). We used the following primary antibodies: rabbit against p-S6 at S235/236 (Cell Signaling Technology 2211S); rabbit against S6 (Cell Signaling Technology 2217S); rabbit against p-eIF2α at S51 (abcam 32157); and rabbit against eiF2α (abcam 242148). The dilution ratios were either 1:1,000 or 1:2,000. Secondary anti-rabbit HRP-linked antibodies (Cell Signaling Technology 7074) were added (dilution ratio: 1:2,000). The signals displayed by the hybridized bands were estimated using GeneTools software (Syngene).

### Short-chain fatty acids concentrations

Short-chain fatty acid concentrations in the cecal contents were quantified after carrying out water extraction (2 vol/wt) and protein precipitation [10% vol/vol phosphotungstic acid (Sigma-Aldrich)] as described elsewhere (48). Briefly, the short-chain fatty acids (SCFAs) in the acidified supernatant (0.3 μl) were separated using a 7890 Gas Chromatography System (Agilent, Les Ulis, France) equipped with a split/splitless injector (ALS7650), a flame-ionization detector, and a Nukol-SP-1000-capillary GC column (15 m × 0.53 nm, 0.5 μm; FSCAP Nukol; Supelco, Saint-Quentin-Fallavier, France). Hydrogen was the carrier gas (flow rate = 10 ml/min). The inlet, column, and detector temperatures were 200, 100, and 240°C, respectively. We used 2-ethylbutyrate (Sigma-Aldrich) as the internal standard. Samples were analyzed in duplicate. The data were collected and the peaks were characterized using OpenLab ChemStation C.01.06 software (Agilent, Les Ulis, France).

### Statistical analysis

All the data were expressed as means ± standard error of the mean (SEM). The survivorship of *C. elegans* was analyzed using the Kaplan–Meier method, and differences among groups were identified using the log-rank test [*survival* and *survminer* packages in R (43)]. The differences among the rat experimental groups were analyzed using a one-way ANOVA followed by Tukey’s tests for *post hoc* analysis (R Studio, Version 1.2.5001, R Studio Inc.). When the data failed to meet the assumption of normality (Shapiro–Wilk normality test), differences among groups were assessed using a Kruskal–Wallis test followed by Dunn’s tests for *post hoc* analysis (R Studio, Version 1.2.5001, R Studio Inc.). For all statistical analyses, the alpha level was 0.05; results were considered to represent a trend (t) if 0.05 < *p* < 0.10.

## Results

### Characteristics of the lactic acid bacteria strains

**FIGURE 2:**
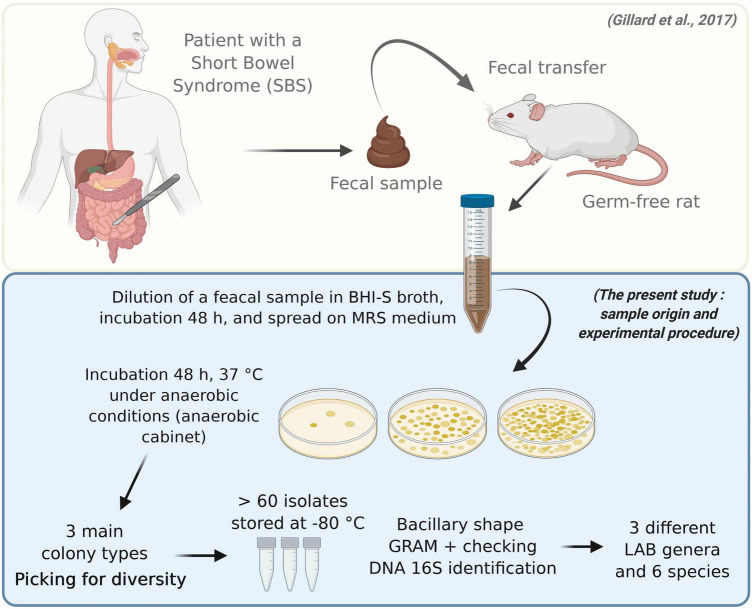
Isolation, characterization, and identification of bacterial strains in the gut microbiota of a patient with short bowel syndrome. Feces from a germ-free rat that had previously been inoculated with fecal microbiota from an SBS patient were collected daily for 3 days and then twice a week for 30 days (37). The fecal sample displayed a stable microbial composition, similar to that of the inoculum. The sample was then incubated in rich, non-selective medium for 48 h, and serial dilutions were plated on MRS medium. The bacteria were incubated for 48 h at 37°C under anaerobic conditions. Various colony types were observed. More than 60 isolates were identified. They represented three genera and five species of lactic acid bacteria: *L. rhamnosus*, *L. casei*, *L. camelliae*, *L. reuteri*, and *L. salivarius*.

Six Gram-positive, bacilliform strains were isolated from the feces of the axenic rats that had been inoculated with the gut microbiota of an SBS patient (Figure 2). Using 16S sequencing, we identified the presence of the following species: *L. casei, Lacticaseibacillus rhamnosus, Lacticaseibacillus camelliae, Limosilactobacillus reuteri*, and *Ligilactobacillus salivarius*.

**FIGURE 3:**
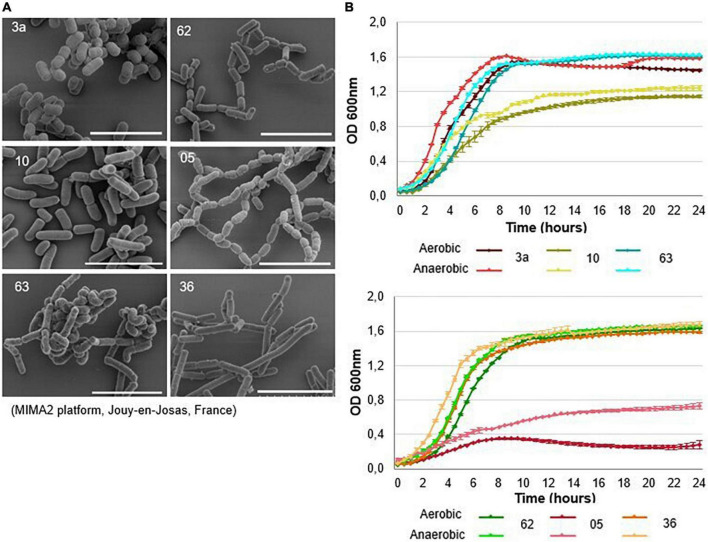
Description of lactic acid bacteria selected for further testing. **(A)** Scanning electron microscopy images and **(B)** growth curves of the six bacterial strains selected. Bacteria were grown in MRS medium under aerobic conditions (dark-colored curves) and anaerobic conditions (light-colored curves) for 24 h at 37°C. Growth was measured using optical density (OD) at 600 nm. Anaerobic conditions were achieved by completely covering the wells with paraffin oil. The mean ± SEM of three wells are shown. The scale bars represent 5 μm.

The strains varied morphologically, with lengths ranging from 1 to 2 μm (Figure 3A). Strains 3a, 36, 62, and 63 showed relatively similar growth patterns under both aerobic and anaerobic conditions; the lag phase lasted about 2 h, and the stationary phase was reached after 8–9 h (Figure 3B). Strain 10 grew more slowly under both aerobic and anaerobic conditions, reaching the stationary phase after 12 h. Strain 05 also displayed more limited growth: its final turbidity value, a proxy for growth, was 0.7 under anaerobic conditions and 0.3 under aerobic conditions, values were twofold and fivefold lower, respectively, than those seen for other strains (Figure 3B). Strains 10 (*L. salivarius*) and 05 (*L. camelliae*) were therefore excluded from further use. We retained strains 3a (*L. reuteri*), 36 (*L. rhamnosus*), 62 (*L. casei*), and 63 (*L. casei*) for further screening.

### Impacts of lactic acid bacteria on *Caenorhabditis elegans*

#### Survival

**FIGURE 4:**
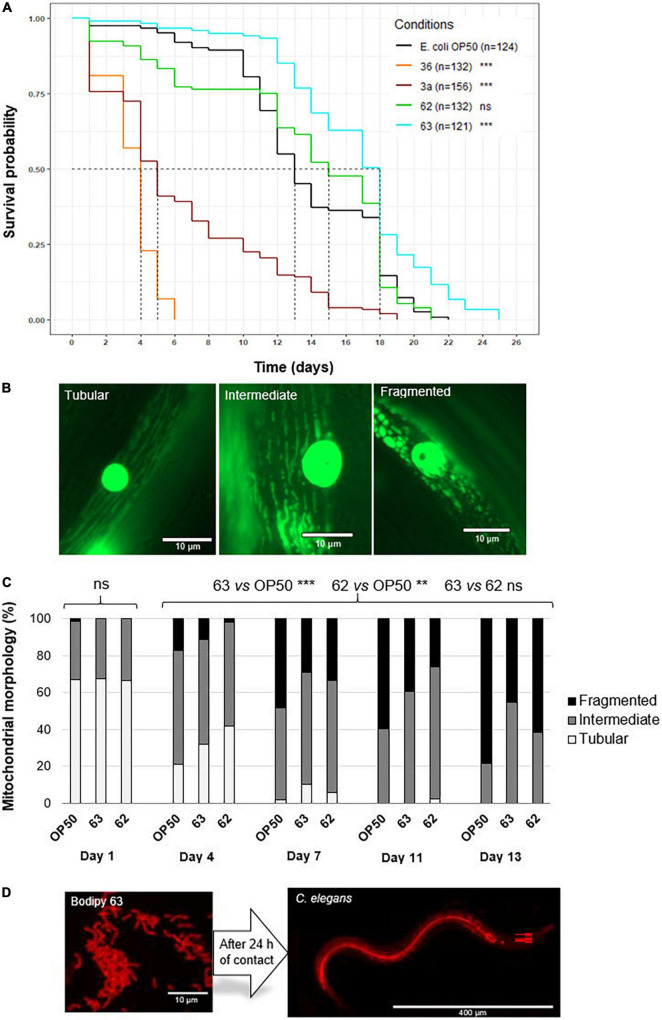
Effect of probiotic supplementation on *Caenorhabditis elegans* survival and body wall muscle structure. **(A)** Median survival of wild-type *C*. *elegans* (N2 strain) fed *E*. *coli* OP50 (control group) or one of the six probiotic strains (treatment groups: 3a, 05, 10, 36, 62, and 63). The asterisks indicate the significance of the comparison between the treatment group and the control group (log-rank test; **p ≤ 0.01; ***p < 0.001; ns: not statistically significant). **(B)** Age-related decline in mitochondrial network structure in *C*. *elegans*. **(C)** Mitochondrial network structure in *C*. *elegans* (PJ1277 strain) fed *E*. *coli* OP50 (*n* = 175 images), probiotic strain 62 (*n* = 76 images), or probiotic strain 63 (*n* = 103 images). Represented are the percentages of nematodes displaying structuration that was tubular (white), intermediate (gray), or fragmented (black). **(D)** BODIPY-stained bacteria in the intestines of *C. elegans* 24 h after ingestion. Significant differences among nematodes over time (day 1, 4, 7, 11, and 13) as well as between the control and the probiotic supplementation groups (strains 62 and 63) were determined using Kruskal–Wallis and Dunn’s *post hoc* tests.

Nematode survival differed among the control and treatment groups (Figure 4A). Nematodes fed strain 63 lived 38.5% longer, on average, than control nematodes (median survival: 18 vs. 13 days, respectively, *p* < 2.10^–16^) and had 13.6% longer total lifespans (maximum survival: 25 vs. 22 days, respectively, *p* < 2.10^–16^). Nematodes given strain 62 displayed equivalent survival to the control nematodes. In contrast, nematodes given strains 3a and 36 had significantly lower median survival than the control (strain 3a: −72.2%, *p* < 2.10^–16^ and strain 36: −77.8%, *p* < 2.10^–16^). They were therefore excluded from further use. These results show that nematode survival was greatly affected by the bacterial strains. Among the LAB tested, only strain 63 significantly improved *C. elegans* survival. Because strain 62 yielded effects similar to those of the control, it was also used in further testing.

#### Mitochondrial networks

In the body wall muscles of the nematodes, mitochondrial network structure was tubular, intermediate, or fragmented (Figure 4B). As also seen in previous research (44, 46), mitochondrial networks became more fragmented as nematodes aged (day 1 vs. day 13, *p* < 0.001) across all treatment groups (Figure 4C). However, in nematodes fed strain 63, this pattern was delayed compared to the control (individuals with fragmented structure at day 13: 45.3 vs. 78.5%, respectively, *p* < 0.001). Nematodes in this treatment group also conserved tubular structure for longer than did the control nematodes (individuals with tubular structure at day 4: 31.9 vs. 21.1%, respectively, *p* < 0.001). Similarly, in nematodes fed strain 62, 61.5% of individuals displayed fragmented structure on day 13, and 41.7% displayed tubular structure on day 4 (*p* < 0.01 when compared with the control). These results suggest that strains 62 and 63 slowed down the aging of nematode muscle cells.

By staining the LAB, we were able to confirm that the nematodes had ingested the bacteria (see Figure 4D) and that the bacteria were able to colonize the gut 24 h after ingestion.

### Effects of probiotic supplementation on “frail” rats

We explored the potential positive impacts of probiotic supplementation with LAB using a model of frailty in rats, where we mimicked a loss of muscle mass induced by decreased food intake, one of the mechanisms responsible for sarcopenia and frailty in elderly humans.

#### Experiment #1: Effects of strain 62 and 63 supplementation on “frail” rats

The AL group ate 29.47 ± 1.42 g of food per day (Table 3). In the R, R+62, and R+63 groups, food intake was 71.3, 71.4, and 70.8% of that in the AL group, respectively. Because the three food-restricted groups had equivalent levels of food intake, any differences observed could be attributed to LAB supplementation. The food-restriction treatment decreased rat body mass (BM) at similar levels in all three groups [−11.9, −13.3, and −15.0% compared to initial BM for the R, R+63, and R+62 groups, respectively; *p* < 0.001 when compared to the AL group (−3.9%)] (Table 3).

**TABLE 3:** Food intake, initial body mass, change in body mass, initial fat mass, change in fat mass, initial lean mass, and change in lean mass in the “frail” rat model during experiment #1.

Parameters	AL group	R group	R+63 group	R+62 group
Food intake (g/day)	29.47 ± 1.42^a^	21.02 ± 0.18^b^	20.87 ± 0.18^b^	21.04 ± 0.16^b^
Initial body mass (g)	615.31 ± 19.62^a^	603.42 ± 21.76^a^	588.07 ± 25.98^a^	617.67 ± 19.82^a^
Body mass change (g)	−23.92 ± 4.86^a^	−71.67 ± 9.72^b^	−78.50 ± 10.90^b^	−92.33 ± 9.63^b^
Initial fat mass (g)	114.23 ± 8.06^a^	126.47 ± 14.88^a^	133.48 ± 13.22^a^	134.53 ± 10.73^a^
Fat mass change (g)	−1.60 ± 3.13^a^	−42.64 ± 6.86^b^	−54.90 ± 8.85^b^	−57.74 ± 7.44^b^
Initial lean mass (g)	471.47 ± 14.76^a^	459.65 ± 11.60^a^	434.85 ± 13.79^a^	465.09 ± 11.18^a^
Lean mass change (g)	−20.95 ± 3.00^a^	−45.57 ± 4.90^b^	−30.37 ± 3.37^a^	−43.00 ± 3.48^b^

Values are means ± SEM. Differences in letters indicate a significant difference between groups (ANOVA and Tukey post hoc tests, *p* ≤ 0.05, n = 54 rats).

In the AL group, fat mass remained stable throughout the experiment (−1.4% of initial fat mass), but lean mass decreased moderately (−4.4% of initial lean mass), which is a normal pattern in older rodents (49, 50). The food-restriction treatment induced a significant loss of both fat and lean mass (R, R+63, and R+62 groups vs. AL group: *p* < 0.05) (Table 3). The loss of fat mass was similar across the three food-restricted groups (final fat mass range: 33.7–42.9% of initial fat mass). Interestingly, lean mass loss was significantly less pronounced in the R+63 group than in the R group (−45.6 ± 5.0 g vs. −30.4 ± 3.4 g respectively, *p* < 0.05) (Table 3), yet it was equivalent in the R+62 and R groups. These results suggest that strain 63 could more efficiently limit lean mass loss in the face of food restriction.

**FIGURE 5:**
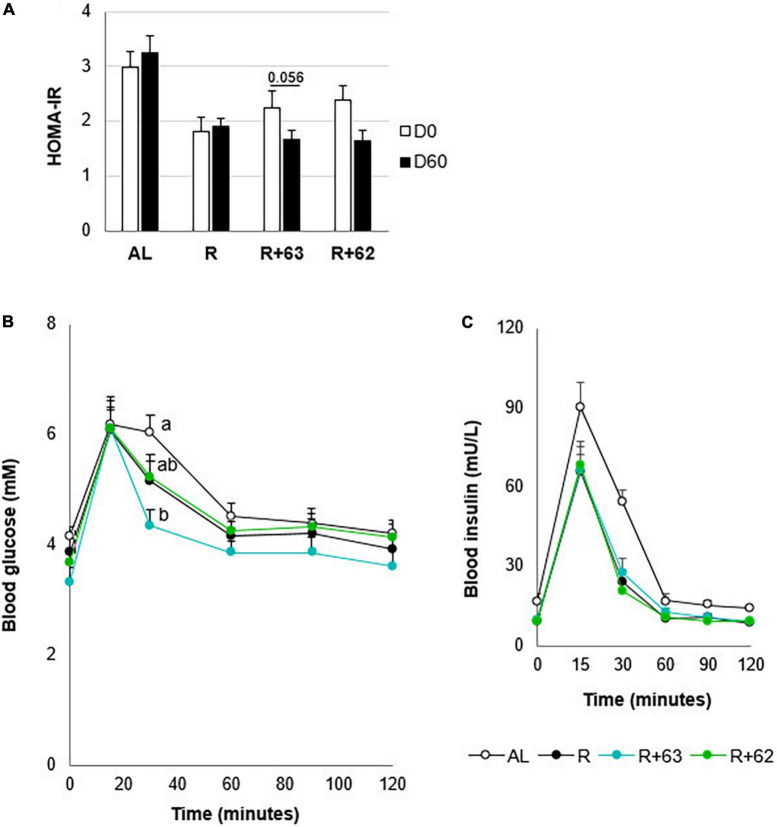
Insulin resistance and glucose tolerance in 18-month-old rats fed an *ad libitum* (AL) diet or a food-restricted (R) diet over a 2-month period; two food-restricted groups received a probiotic supplement – either strain 62 (R+62) or strain 63 (R+63). **(A)** Insulin resistance at the start (day 0) and end (day 60) of the experiment. **(B)** Plasma glucose levels measured during the oral glucose tolerance test on day 60. **(C)** Plasma insulin levels during the oral glucose tolerance test on day 60. Values are means ± SEM. Differences in letters indicate a significant difference between groups (ANOVA and Tukey *post hoc* tests, *p* ≤ 0.05, *n* = 54 rats).

The experimental groups differed in their insulin resistance at days 0 and 60 (Figure 5A). Because the rats went through a 15-day dietary acclimation period before the experiment began (i.e., food restriction for the R, R+62, and R+63 groups), the three food-restricted groups had 46.8% lower insulin resistance than the AL group at day 0 (*p* < 0.05). Between days 0 and 60, insulin resistance decreased even further in the R+63 group (*p* = 0.056) but not in the R or R+62 groups (*p* > 0.1 in both). Of note, this difference stems from the fact that, relative to the R group, the R+63 group had slightly, but not significantly, higher insulin resistance at day 0 and slightly lower insulin resistance at day 60. Furthermore, on day 60, oral glucose tolerance at 30 min was 26.5% lower in the R+63 group than in the AL group (4.7 ± 0.4 vs. 6.4 ± 0.5 mM, *p* = 0.007) (Figure 5B). The four groups did not differ significantly in the blood glucose levels (iAUC) measured 1 h after glucose ingestion (data not shown). Insulin levels were identical in the R, R+62, and R+63 groups (Figure 5C). These findings suggest that strain 63 could increase insulin sensitivity at the whole-body level.

At the end of the experiment, levels of glucose, lactate, urea, HDL cholesterol, LDL cholesterol, and total cholesterol did not differ among the four groups (Supplementary Figure 1).

#### Experiment #2: Mechanistic action of strain 63 supplementation in rats

##### Muscle protein synthesis and breakdown

Because it was the only strain with beneficial effects in the first experiment, strain 63 was the focus of the second, 1-month experiment exploring the bacterium’s mechanisms of action. The study duration was shortened because, during the first experiment, the 2 months of food restriction led to pronounced rat mortality.

As in the first experiment, food intake was 29% lower in both the R and R+63 groups compared to the AL group (*p* < 0.05) (Table 4). The body mass of the AL group remained fairly stable throughout the experiment. Food restriction led to an equivalent decline in body mass in both treatment groups (−5.9 and −5.4% for R and R+63, respectively, *p* < 0.05 compared to AL) (Table 4). This pattern resulted from the greater loss of both fat and lean mass in the food-restricted groups (R and R+63 vs. AL, *p* < 0.05), with the R+63 group losing relatively more fat mass (R+63 vs. R and AL, *p* < 0.05). Although the R group lost more absolute lean mass than did the R+63 group, the difference was not statistically significant (*p* > 0.1), perhaps because of the experiment’s shorter duration.

**TABLE 4:** Food intake, initial body mass, change in body mass, initial fat mass, change in fat mass, initial lean mass, and change in lean mass in the “frail” rat model during experiment #2.

Parameters	AL group	R group	R+63 group
Food intake (g/day)	29.12 ± 0.30^a^	20.61 ± 0.03^b^	20.69 ± 0.05^b^
Initial body mass (g)	563.47 ± 12.93^a^	525.92 ± 8.00^a^	532.73 ± 8.83^a^
Body mass change (g)	7.53 ± 3.93^a^	−31.08 ± 3.83^b^	−28.55 ± 2.85^b^
Initial fat mass (g)	82.32 ± 5.06^a^	68.32 ± 3.56^b^	71.61 ± 3.65^b^
Fat mass change (g)	10.91 ± 2.44^a^	−6.30 ± 1.43^b^	−10.14 ± 1.41^c^
Initial lean mass (g)	439.41 ± 11.30^a^	415.78 ± 6.13^a^	420.99 ± 9.71^a^
Lean mass change (g)	−1.18 ± 2.79^a^	−19.18 ± 2.22^b^	−14.02 ± 2.40^b^

Values are means ± SEM. Differences in letters indicate a significant difference between groups (ANOVA and Tukey post hoc tests, *p* ≤ 0.05, n = 61 rats).

**FIGURE 6:**
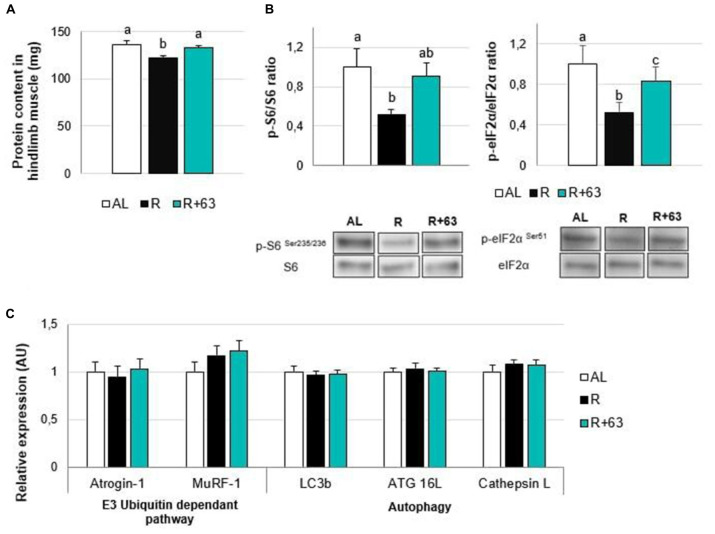
Levels of protein, insulin pathway mediators, and proteolysis-related gene expression in the muscles of 18-month-old rats fed an *ad libitum* (AL) diet or a food-restricted (R) diet over a one-month period; one food-restricted group received a probiotic supplement – strain 63 (R+63). **(A)** Protein levels in hindlimb muscles (gastrocnemius, soleus, tibialis anterior, and extensor digitorum longus). **(B)** Levels of proteins involved in the AKT/mTOR/S6K pathway: ratio of phosphorylated proteins, p-S6 (Ser235/236), and p-eIF2α (Ser51), to total proteins, S6 and eIF2α. **(C)** Expression levels of muscle proteolysis genes, namely those related to the regulation of the ubiquitin-proteasome-dependent pathway (Atrogin-1 and MuRF-1) and autophagy pathway (Lc3b, ATG16L, and Cathepsin L). Values are means ± SEM. Differences in letters indicate a significant difference between groups (Kruskal–Wallis test and Dunn’s *post hoc* test, *p* ≤ 0.05, *n* = 61 rats).

However, strain 63 seemed to help preserve protein synthesis in the hindlimb muscles, given that protein levels were 8.0% higher in the R+63 group than in the R group (*p* = 0.02) but similar between the R+63 and AL groups (*p* = 0.76) (Figure 6A).

Losses of muscular protein mass could be explained by the altered activation of the mTOR signaling pathway: compared to the AL group, the R group’s p-S6 phosphorylation levels were 47.9% lower (*p* < 0.05), and its p-eIF2α levels were 47.6% lower (*p* < 0.01) (Figure 6B). Interestingly, the p-S6/S6 and p-eIF2α/eIF2α ratios in the R+63 group were 90.3 and 83.5% of those in the AL group, respectively, indicating that they were partially maintained. The R+63 group occupied an intermediate position: its p-S6/S6 ratio was similar to those of the AL and R groups, but its p-eIF2α/eIF2α ratio was not. This result suggests that, when food is restricted, strain 63 could limit muscle atrophy by maintaining the Akt/mTOR/S6 protein synthesis-related pathway within muscles. With regards to protein breakdown, neither food restriction nor strain 63 supplementation affected the expression of genes involved in the ubiquitin proteasome pathway (Atrogin-1 and Murf-1) or autophagy (Lc3b, ATG 16L, and Cathepsin L) (Figure 6C).

##### Hindgut

**FIGURE 7:**
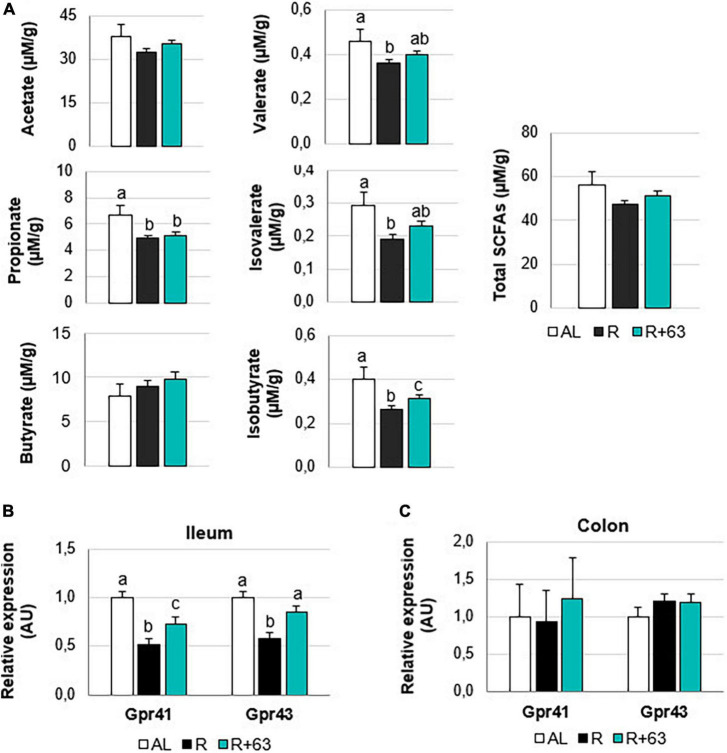
Short-chain fatty acids levels in the cecal contents and expression of GPR41 and GPR43 in the colon and ileum of 18-month-old rats fed an *ad libitum* (AL) diet or a food-restricted (R) diet over 1-month period; one food-restricted group received a probiotic supplement – strain 63 (R+63). **(A)** Levels of the SCFAs acetate, propionate, butyrate, valerate, isovalerate, and isobutyrate in the cecal contents of the R and R+63 groups. **(B)** GPR41 and GPR43 expression in the distal ileum. **(C)** GPR41 and GPR43 expression in the proximal colon. Values are means ± SEM. Differences in letters indicate a significant difference between groups (Kruskal–Wallis test and Dunn’s *post hoc* test, *p* ≤ 0.05, *n* = 61 rats).

Food restriction significantly decreased cecal levels of propionate (−26.9%), valerate (−21.7%), isovalerate (−34.5%), and isobutyrate (−32.5%) (R vs. AL, *p* < 0.05) (Figure 7A). Only butyrate was unaffected. Valerate, isovalerate, and isobutyrate concentrations tended to be higher in the R+63 group than in the R group (+8.7, +13.8, and +10.0%, respectively, *p* = 0.1). SCFAs interact with specific gut receptors (GPRs), whose expression within the hindgut can be measured. Here, groups did not differ in GPR expression within the colon (Figure 7C). That said, levels of mRNA for GPR43 and GPR41 were, respectively, 1.7-fold and 1.9-fold lower in the R group compared to the AL group (*p* < 0.05) (Figure 7B). In the R+63 group, GPR43 expression was equivalent to that in the AL group; GPR41 expression was somewhat but not entirely similar.

## Discussion

Elderly people often experience a loss of appetite, decreased nutrient absorption, and/or a decline in metabolic efficiency. Such can put them at greater risk of sarcopenia, and they may enter into a state of frailty/dependence. In this study, we started with the gut microbiota of an individual with SBS, because, within the months following intestinal resection surgery, most patients have been found to compensate behaviorally, physiologically, and metabolically, eventually restoring effective nutrient absorption (51). These responses include hyperphagia, gut wall hyperplasia, and increased secretion of gut hormones, and they occur in tandem with changes in gut microbiota composition. Research has shown that the microbiota of SBS patients is quite different from what is seen in “healthy” individuals and is mainly composed of aerobic lactobacilli (36, 37). However, fecal transplants of this microbiota to axenic rats resulted in higher levels of GLP-1, ghrelin, and leptin, as well as the presence of deeper intestinal crypts (37). This research suggested that the SBS microbiota could prompt physiological and metabolic responses that improve nutrient bioavailability. Consequently, we hypothesize that certain members of the SBS microbiota could improve nutrient availability and anabolic effects in elderly people with decreased food intake and sarcopenia. Here, we showed that when one of these bacteria, *L. casei* CNCM I-5663 (strain 63), was ingested daily for 1 month, food-restricted rats were able to preserve muscle protein mass by improving both protein synthesis within the muscle tissue and insulin sensitivity within the muscle and whole body.

Research has already been exploring the use of human microbiota in probiotic treatments. To date, the focus has been on comparing the effects of gut microbiota from people with “healthy” phenotypes vs. “unhealthy” phenotypes. This work has been based on the assumption that a “healthy microbiota” is enriched in certain bacterial genera/species (i.e., symbionts) that have health benefits whereas an “unhealthy” microbiota lacks these same bacteria. For example, in humans, when the relative abundance of *Akkermansia muciniphila* is higher, the risk of metabolic syndrome is lower (52, 53). *Akkermansia* supplementation is effective in limiting the occurrence of metabolic disorders in both rodents (54) and humans (55). *Faecalibacterium prausnitzii* shows promise as a probiotic because of its anti-inflammatory effects (56, 57). On this same note, clinical work has found that levels of *F. prausnitzii* were lower in people with chronic inflammatory bowel disease vs. healthy individuals (58).

In contrast, the SBS microbiota is extremely different from the above “healthy” microbiota because it is 90% composed of LAB (mainly the genera *Lactobacillus*, *Lacticaseibacillus*, and *Limosilactobacillus*), a group with less than 1% representation in “healthy” individuals (36). To explore the potential probiotic properties of interest to us within this atypical microbiota, we employed an additional screening step that exploited a useful *in vivo* model, the nematode *C. elegans* (59, 60). Various screening parameters can be used with this model: colonization of the intestine, survival rate, oxidative stress, and levels of proteins or genes involved in metabolic pathway regulation (61–63). In probiotics testing, the *E. coli* strain OP50 is generally used as the control, as was the case here. In our study, we used survival as a key screening parameter. We also used a *C. elegans* mutant from the Caenorhabditis Genetics Center that can be exploited to highlight muscle structure and function (60). With GFP-labeled nematodes, it is possible to visualize sarcomeres and mitochondria in the muscle tissue *via* fluorescence microscopy (64). Strains 62 and 63 maintained the muscle mitochondrial network in a less-fragmented state over time compared to control worms, suggesting that they had a positive effect on muscle integrity. Furthermore, nematode survival was maintained (strain 62) and even increased (strain 63) compared to OP50-fed worms. Because of these promising results, strains 62 and 63 were then tested in a model of frail rodents.

Our model consisted of elderly rats (18 months) subject to a moderate level of food restriction, which imitates a situation in which nutrient availability is limited, prompting a decrease in muscle mass. We showed that strain 63 helped preserve lean mass in the face of food restriction during both the 2-month and 1-month experiments. In contrast, we saw no tangible benefits arising from strain 62. Over recent years, several studies have tested the health effects of probiotic bacteria in elderly animals [see Giron et al. (29) for a recent review]. To our knowledge, none of this research has applied conditions of food restriction, even though lower food intake contributes to sarcopenia and frailty in elderly people (1, 65–69). Indeed, it has been estimated that those are at greatest risk are those whose energy and protein intake are at 75–80% of recommended levels (energy: 1,300–1,600 kcal/day for women and 1,400–1,900 kcal/day for men (70–72); protein: 0.8 g/kg of body mass/day (68)). The rats in our model were therefore fed 70–75% of the quantity consumed by rats on an *ad libitum* diet.

A few studies have investigated microbiota–gut–muscle interactions in rodents. They have shown that probiotic supplementation regimes lasting from 3 to 12 weeks can positively influence muscle mass and/or function (e.g., grip strength, running time, and markers of fatigue) in young mice (21, 22, 73), rats (23, 24), or SAMP8 mice, a model of accelerated aging (30–32). There is even less research examining how probiotic supplementation impacts muscle mass and function in elderly humans. The single study performed to date found that *Lactobacillus plantarum* TWK10 had beneficial effects on muscle mass and strength in frail elderly people (74). Our study has confirmed that microbiota-based treatments could help limit sarcopenia in this population. Additionally, we would argue that our approach could be effective in challenging nutritional contexts in which dietary supplementation strategies would be difficult to implement. Finally, our results have shed light on the mechanisms by which the probiotic is likely acting.

First, we found that probiotic supplementation improved insulin sensitivity at the whole-body level and enhanced glucose tolerance. This discovery fits with past work showing that the gut microbiota in general, and certain bacteria and metabolites in particular, may be involved in glucose homeostasis (75). Indeed, alterations may underlie the development of metabolic disorders and insulin resistance [for a review, see Régnier et al. (76)]. Additionally, recent studies on insulin-resistant rodents have shown that probiotic supplementation (mainly with *Bacillus*, *Lactobacillus*, *Bifidobacterium*, and *Akkermansia* species) could dampen insulin resistance (77), a phenomenon that could be partially explained by changes in the microbial metabolites (i.e., SCFAs) interacting with gut epithelial cells. Here, we did not see an increase in cecal SCFA levels following probiotic supplementation, except in the case of minor SCFAs (isobutyrate and, to a lesser extent, valerate and isovalerate). However, within the ileum, there was an escalation in the expression of two insulin-sensitizing receptors (GPR41 and GPR43). This observation suggests that strain 63 improved insulin sensitivity by boosting receptor expression instead of SCFA production. It is possible to link the effects of strain 63 on GRP 41 and 43 to the increased secretion of incretin and, in particular, of GLP-1 (37, 39), whose secretory cells are located in the distal ileum (78). Contrary to previously tested probiotics, strain 63 did not appear to alter inflammatory targets, such as TNF-α, in the ileum and muscle tissues (Supplementary Figure 2). The effects we saw seemed to be independent of intestinal barrier integrity and permeability. Mechanistically, by increasing insulin sensitivity, strain 63 could positively influence protein metabolism in the muscles and promote a stronger anabolic response, which could, in turn, explain the better maintenance of muscle protein mass.

Muscle protein mass results from the balance between muscle protein synthesis and proteolysis, an interplay that is shaped by the presence of dietary amino acids and insulin and that can become dysregulated during aging (4, 7, 79). In this study, the food-restriction treatment lowered amino acid availability. Furthermore, strain 63 likely acted on the gut-muscle cross-talk by mediating insulin and its signaling pathway, thus affecting protein synthesis and proteolysis (i.e., the Akt/mTOR pathway). It seems unlikely that the strain operated by inhibiting muscle protein breakdown *via* the ATP-ubiquitin proteasome and autophagy-lysosome systems, which are the two main forces behind muscle protein degradation (80). This result contrasts with those of previous studies in which probiotic supplementation was found to inhibit proteolysis. This difference probably arises from the fact that such studies used models simulating situations of dramatic inflammation, such as cancer cachexia (81, 82). In our “frail” rat model, no increase was seen in either the markers of muscle proteolysis (mRNA for Atrogin-1, Murf-1, Lc3b, ATG16L, and Cathepsin L in gastrocnemius muscle) or the markers of inflammation (mRNA for TNF-α in the ileum and muscle tissue) in the non-supplemented food-restricted group (Supplementary Figure 2). These findings concur with those of past research: a dietary restriction treatment similar to ours had no effect on the expression of the muscle atrophy markers, Atrogin-1 and Murf-1 (83–85). It seems probable that further research using a different model of frailty (i.e., one that is not based on food restriction) is needed to better explore how muscle proteolysis might be mediated by atrogenes. Here, it was apparent that probiotic supplementation functioned by increasing muscle protein synthesis: the treatment maintained the phosphorylation levels of proteins that help regulate protein synthesis *via* mTOR activation (S6 and eIF2α). The mTOR pathway is a strong driver of insulin-mediated protein synthesis (80), which can explain why probiotic supplementation had a long-term impact on muscle protein mass. Indeed, previous studies have found that probiotics can have stimulatory effects on mTOR pathway components (Akt, S6K, and mTOR) in both cachectic muscles (81) and non-cachectic muscles (23).

## Conclusion

In conclusion, using our model of frail rodents, we discovered that supplementation with *L. casei* 63 could limit muscle loss due to age and food restriction. Mechanistically, the strain acted by improving insulin sensitivity at the whole-body level and within muscles, which led to the maintenance of protein synthesis. In contrast to other probiotics we have tested, whose target is the immune response (Patent WO2020020540A1), *L. casei* 63 directly operates on protein anabolism along the gut–muscle axis *via* the insulin response, which is independent of inflammation status. This strain thus shows promise, and its use as a complementary tool for preventing sarcopenia in the elderly should be considered in the future. Indeed, *L. casei* 63 supplementation could be effective in individuals for which common strategies such as protein supplementation or physical exercise have failed.

## Data availability statement

The original contributions presented in this study are included in the article/Supplementary material, further inquiries can be directed to the corresponding author.

## Ethics statement

The animal study was reviewed and approved by the Animal Care and Use Committee of Auvergne (CEMEA Auvergne; Permit Number: C2EA-02) and the Ministry of Higher Education and Research.

## Author contributions

IS-A, CC, and MT came up with the main research ideas. CM, MG, and CC isolated and identified the bacteria strains. MG performed the experimental work on *C. elegans*. IS-A, MJ, and DD designed the experimental work on rats. IS-A, MJ, MG, DD, and CC collected samples from the rats. MG and MJ performed laboratory analyses on the samples. M-LN, GF, and CM provided technical support with the MS-GC and spectrophotometry. MG and IS-A carried out the data analysis and the statistical analysis, created the tables/figures, and wrote the manuscript. DD and CC helped revise and improve the manuscript. All authors have read the manuscript and agreed to its submission.
