# Corrigendum: *Lacticaseibacillus casei* CNCM I-5663 supplementation maintained muscle mass in a model of frail rodents

**DOI:** 10.3389/fnut.2022.1109835

**Published:** 2023-01-04

**Authors:** Muriel Giron, Muriel Thomas, Marianne Jarzaguet, Camille Mayeur, Gladys Ferrere, Marie-Louise Noordine, Stéphanie Bornes, Dominique Dardevet, Christophe Chassard, Isabelle Savary-Auzeloux

**Affiliations:** ^1^INRAE, UMR 1019, Unité de Nutrition Humaine, Université Clermont Auvergne, Clermont-Ferrand, France; ^2^Université Paris-Saclay, INRAE UMR 1319, AgroParisTech, Micalis Institute, Jouy-en-Josas, France; ^3^INRAE UMR 0545, Unité Mixte de Recherche sur le Fromage, Université Clermont Auvergne, VetAgro Sup, Aurillac, France

**Keywords:** muscle, sarcopenia, probiotic, lactic acid bacteria, insulin sensitivity, protein synthesis

In the published article, there was an error in the legend for Figure 6A as published. The title on Y-axis is “Protein content in hindlimb muscle (mg/g).” The corrected legend appears below.

**Figure 6:**
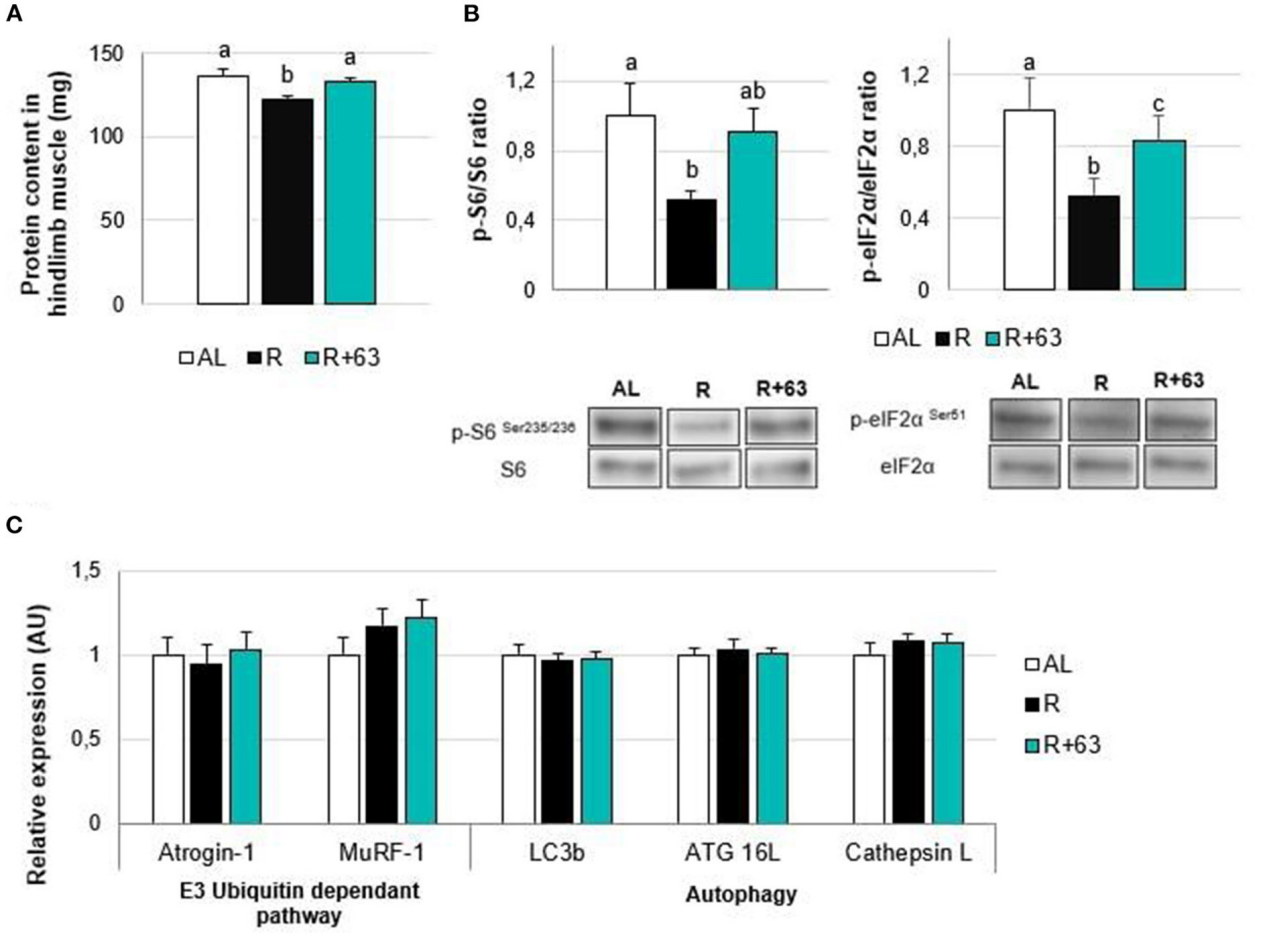
Levels of protein, insulin pathway mediators, and proteolysis-related gene expression in the muscles of 18-month-old rats fed an ad *libitum* (AL) diet or a food-restricted (R) diet over a one-month period; one food-restricted group received a probiotic supplement – strain 63 (R+63). **(A)** Protein levels in hindlimb muscles (gastrocnemius, soleus, tibialis anterior, and extensor digitorum longus). **(B)** Levels of proteins involved in the AKT/mTOR/S6K pathway: ratio of phosphorylated proteins, p-S6 (Ser235/236), and p-eIF2α (Ser51), to total proteins, S6 and eIF2α. **(C)** Expression levels of muscle proteolysis genes, namely those related to the regulation of the ubiquitin-proteasome-dependent pathway (Atrogin-1 and MuRF-1) and autophagy pathway (Lc3b, ATG16L, and Cathepsin L). Values are means ± SEM. Differences in letters indicate a significant difference between groups (Kruskal–Wallis test and Dunn's post hoc test, p ≤ 0.05, n = 61 rats).

The authors apologize for this error and state that this does not change the scientific conclusions of the article in any way. The original article has been updated.

