# Supplementary material for: Lacticaseibacillus casei CNCM I-5663 supplementation maintained muscle mass in a model of frail rodents
Source: Front Nutr. 2022 Aug 10;9:928798. doi: 10.3389/fnut.2022.928798 (PMC9399775; doi:10.3389/fnut.2022.928798)
Supplement: Supplementary file 1 [file Data_Sheet_1.DOCX]

**Supplementary material**

**(A)**

**(C)**

**(B)**

**(D)**

**Figure S1. Gut hormones and plasma biochemical parameters measured in 18-month-old rats following 2 months of food restriction (R) ± supplementation in strain 63 (R+63) or 62 (R+62). (A)** Plasma GLP-1 concentration measured in the portal vein 1 h after food intake (fed state). **(B)** Plasma GLP- 1 concentration measured in the tail artery in the fasted state. **(C)** Plasma PYY concentration measured in the portal vein 1 h after food intake (fed state). **(D)** Plasma arterial concentration of urea, total cholesterol, triglycerides (TAG), LDL-cholesterol, HDL-cholesterol, glucose and lactate 1h after food intake (fed state).

Data are presented as mean ± SEM (n = 54 rats). No significant difference between AL, R, R+63 and R+62 p ≤ 0.05 (ANOVA).

**(B)**

**(A)**


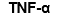

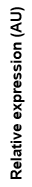


**Figure S2. mRNA levels of Inflammation parameters in 18-month-old rats following 1 months of food restriction (R) ± supplementation in strain 63 (R+63). (A)** mRNA levels of TNF-α and IL-10 in ileum. **(B)** mRNA levels of TNF-α in gastrocnemius muscle.

Data are presented as mean ± SEM (n = 61 rats). No significant difference between AL, R and R+63 p ≤ 0.05 (ANOVA).
